# An empirical study on the relationship between state anxiety, acute mountain sickness, oxygen saturation, and rating of perceived exertion among graduate students during high-altitude mountaineering

**DOI:** 10.3389/fpsyg.2026.1754190

**Published:** 2026-02-26

**Authors:** Yuanbin Sang, Xiaolong Wang, Jiahao Jiang, Zhengyang Zeng, Yating Huang, Lun Li

**Affiliations:** 1 School of Physical Education, China University of Geosciences, Wuhan, China; 2 School of Public Administration, China University of Geosciences, Wuhan, China

**Keywords:** acute mountain sickness, graduate students, high-altitude mountaineering, rating of perceived exertion, state anxiety

## Abstract

**Background:**

In recent years, as participation in high-altitude mountaineering has expanded, high-altitude climbing has increasingly become a challenging form of practical activity among university students, particularly among graduate students who experience high levels of academic and research-related stress. However, the physiological and psychological responses of this population in high-altitude environments remain insufficiently examined by systematic empirical research.

**Objective:**

This study examined changes in state anxiety (STAI-Y1) before and after a 3-day high-altitude mountaineering expedition at 2,726–5,396 m and analyzed its relationships with acute mountain sickness (AMS), arterial oxygen saturation (SpO₂), and rating of perceived exertion (RPE) in graduate students. Additionally, it aimed to provide empirical evidence to help identify high-risk individuals and improve pre-ascent preparation and psychological support in high-altitude mountaineering.

**Methods:**

A total of 15 graduate students were recruited for a 3-day mountaineering expedition. The STAI-Y1 was administered before and after the climb. During the ascent, SpO₂ was measured using a portable finger pulse oximeter, AMS was assessed using the Lake Louise Acute Mountain Sickness Score (LLS), and subjective fatigue was recorded via the Borg 6–20 RPE scale. Data on AMS and summit success were documented throughout the journey. Paired t-tests, between-group comparisons, and multiple linear regression were employed to analyze post-climb STAI-Y1 scores.

**Results:**

Following the climb, LLS scores increased from 0 to 5.7, SpO₂ decreased from 94 to 88%, and RPE rose from 6 to 19 (*p* < 0.001). In contrast, STAI-Y1 scores showed a non-significant decrease from 46.5 ± 12.9 to 43.9 ± 14.1 (*p* = 0.603). STAI-Y1 scores were higher in the AMS group and in non-summiters than in their respective comparison groups (*p* < 0.05). Multiple regression analysis indicated that pre-climb STAI-Y1 (*β* = 0.916) and the change in RPE (ΔRPE; B = 2.798) were significantly positively associated with post-climb STAI-Y1, whereas the change in SpO₂ (ΔSpO₂) was not significant.

**Conclusion:**

Short-term high-altitude mountaineering imposed considerable physiological strain and induced AMS symptoms, yet overall state anxiety did not increase significantly. Increases in RPE were also significantly associated with changes in state anxiety, whereas changes in SpO₂ had only a limited impact on state anxiety.

## 1 Introduction

Graduate students constitute a highly educated, high-pressure population exposed to sustained academic and research demands. As a result, they tend to show elevated levels of anxiety (Chi et al., 2023). Empirical studies indicate that 15–25% of graduate students report dissatisfaction with their academic experience (Woolston, 2022). Furthermore, 41 and 39% meet criteria for depression and anxiety, respectively (Evans et al., 2018), highlighting anxiety as a major threat to both academic persistence and mental health (Liu, 2020; Satinsky et al., 2021). Additional evidence suggests that graduate students are already facing a mental health crisis. In recent years, graduate students have increasingly participated in high-altitude mountaineering activities (Xiang et al., 2024), a form of extreme physical activity that imposes substantial physiological and psychological demands.

Climbing mountains above 2,500 m exposes individuals to hypobaric hypoxia and cold environments, conditions that elicit pronounced physiological responses and increase the risk of acute mountain sickness (Wei et al., 2021; Zeng et al., 2024; Zhang et al., 2024). These environmental stressors may also disrupt emotional and cognitive functioning (Bian et al., 2015; Figueiredo et al., 2022). However, it remains unclear whether such extreme conditions exacerbate or alleviate anxiety among non-professional graduate student climbers (Bian et al., 2015; Figueiredo et al., 2022). In this context, state anxiety refers to a transient condition of tension and apprehension elicited by perceived threat (Smith, 2013). The Borg 6–20 rating of perceived exertion (RPE) provides a subjective measure that closely reflects physiological load (Scherr et al., 2012). Importantly, the Lake Louise Score (LLS) offers a standardized criterion for the assessment of AMS (Roach et al., 2018), and higher RPE values during high-altitude mountaineering have been associated with more severe AMS symptoms (Mellor et al., 2014; Hazlerigg et al., 2016). In parallel, arterial oxygen saturation (SpO₂) declines with increasing altitude, indicating the presence of hypoxic burden (Hackett et al., 1976; Wei et al., 2021). Taken together, state anxiety, RPE, SpO₂, and AMS represent key psychological and physiological dimensions of the high-altitude mountaineering experience.

Among various intervention pathways, physical activity has been shown, under certain conditions, to alleviate anxiety symptoms (Aylett et al., 2018; Kandola et al., 2018; Herbert et al., 2020; Singh et al., 2023). However, high-altitude mountaineering differs substantially from laboratory-based or recreational exercise paradigms, as it combines prolonged physical exertion with environmental stressors and meteorological uncertainty (Chen et al., 2022). Field-based research has suggested that university students may exhibit reduced state anxiety following the completion of a mountaineering route (Castanier et al., 2011). In contrast, elevated anxiety levels have also been reported in association with the onset or exacerbation of AMS symptoms (Hüfner et al., 2022; Tang et al., 2023), highlighting the complex interplay between emotional and physiological processes in high-altitude environments.

Although previous studies have independently examined the effects of high-altitude mountaineering on emotional states or explored associations between perceived exertion and acute high-altitude responses, this evidence remains largely fragmented and insufficient to capture the concurrent dynamics of multiple psychological and physiological factors during real-world high-altitude mountaineering.

As far as we are aware, very few studies in high-altitude mountaineering settings have simultaneously tracked state anxiety, perceived exertion and high-altitude physiological responses, and virtually none have provided systematic empirical evidence in samples of graduate student mountaineers. In addition, much of the existing evidence is derived from laboratory-based hypoxia paradigms, and direct field evidence capturing concurrent changes in anxiety, rating of perceived exertion (RPE), and physiological responses during real-world high-altitude mountaineering remains limited. The present study is innovative in two main respects: first, it examines whether short-term high-altitude mountaineering exacerbates or alleviates state anxiety in graduate students; second, it evaluates the extent to which pre-climb anxiety, changes in perceived exertion during the ascent and factors such as AMS are related to anxiety levels at the end of the climb. By examining state anxiety, RPE, and physiological indicators during an actual high-altitude mountaineering expedition, the present study provides empirical evidence in a population of non-professional graduate student climbers, thereby addressing this gap in the literature.

## 2 Methods

### 2.1 Study design

#### 2.1.1 Location

From 15 to 17 July 2025, a 3-day mountaineering expedition (ascent and descent, approximately 28 km in total) was carried out on Mount Haba (5,396 m) in Shangri-La County, Diqing Tibetan Autonomous Prefecture, Yunnan Province, China. The region is characterised by highly variable weather and large diurnal temperature differences and represents a transition zone from a plateau monsoon climate to a cold temperate mountain climate (Table 1).

**Table 1:** The trekking itinerary of Haba Snow Mountain.

Day	Segment	Start altitude (m)	End altitude (m)	Altitude change (m)	Activity
Base	Wuhan–Lijiang	23	2,400	+2,377	Transport
D1	Lijiang–Haba Village	2,400	2,726	+326	Rest
D2	Haba Village–Haba base camp	2,726	4,100	+1,374	Trekking ascent
D3	Base camp–Haba summit–Haba Village	4,100	2,726	−1,374	Trekking ascent and descent

#### 2.1.2 Low-altitude baseline measurements

Before the ascent, all student participants underwent a medical examination at a designated hospital, and only those who were medically fit were included in the study. On 6 July, participants were informed about the study procedures, and on 7 July they took part in a simulated data-collection session. On the day before the climb, they completed the State–Trait Anxiety Inventory (STAI, Y form) at bedtime in Lijiang (approximately 2,400 m) to measure baseline anxiety.

#### 2.1.3 Measurement during mountaineering

During the climb, we used the Lake Louise Score (LLS) and the Borg Rating of Perceived Exertion scale (RPE, 6–20 points) to measure acute mountain sickness symptoms and perceived fatigue, and we simultaneously measured arterial oxygen saturation (SpO₂). SpO₂, RPE and LLS were measured approximately 30 min after waking each morning, at the highest altitude reached that day, and in the evening after all group activities had finished. On day 1, measurements were taken at 2,400 m and 2,726 m; on day 2 at 2,726 m, 3,718 m and 4,100 m; and on day 3 at 4,100 m, at each participant’s highest altitude reached that day, and again at 2,726 m. SpO₂ was measured using a portable fingertip pulse oximeter (PHILIPS, DB 12, Suzhou Erda Medical Equipment Co., Ltd., China). Because cold exposure at high altitude may bias peripheral oxygen saturation readings, participants were seated quietly indoors for 5 min before each measurement, with both hands placed in clothing pockets or kept warm by wearing gloves. Measurements were taken three consecutive times, and the mean value was used for analysis. RPE was assessed three times per day, and the highest value reported each day was retained to represent the participant’s maximum perceived exertion for that day.

On day 1 at 13:00, participants set off with light packs from Lijiang, Yunnan (2,400 m) and arrived at Haba Village (2,726 m) at 15:00, where they stayed overnight. On day 2, they departed at 07:00, reached an intermediate station at 3,718 m at 11:00, and arrived at the Mount Haba base camp (4,100 m) at 15:50, where they spent the second night. On day 3 at 03:00, they set out to ascend the main peak of Mount Haba (5,396 m). During the descent, they returned first to the 4,100 m base camp for food and rest and then continued down to Haba Village. The daily vertical ascent was controlled within 1,200–1,400 m, and the total round-trip duration did not exceed 16 h. Although it was possible to ride mules up to base camp, all participants completed exactly the same route on foot with light packs.

#### 2.1.4 Post-climb state anxiety measurements

On day 3, upon returning to Haba Village (2,726 m), participants completed the STAI-Y1 state anxiety scale at 19:00 to measure their anxiety levels after the climb.

#### 2.1.5 Symptom and behavioural recording

Three trained assessors accompanied the team and recorded summit success and the occurrence of AMS for each participant. During the ascent, we continuously monitored climbers for signs of high-altitude illness and, when necessary, advised rest or descent to control potential risk. In this study, STAI-Y1 and RPE were measured using electronic questionnaires on the Wenjuanxing platform, self-administered by participants under on-site supervision by the assessors, whereas LLS was measured using paper forms completed by the assessors. All scales were formally implemented after a small pilot test and standardised training of the investigators prior to departure.

### 2.2 Participants

Participants were recruited on a voluntary basis from graduate students at China University of Geosciences (Wuhan). Inclusion criteria were non-professional graduate student mountaineers who were in good physical health, with no history of smoking or substance dependence, no prior altitude-related illnesses, cardiopulmonary diseases, or musculoskeletal injuries that could affect mountaineering performance, and no exposure to high-altitude environments within the previous 6 months. A total of 15 graduate student mountaineers were included in the study, comprising 12 master’s students and 3 doctoral students. The participants had a mean age of 28 ± 8 years, a mean height of 176 ± 11 cm and a mean body mass of 71.3 ± 11.5 kg (Table 2). No monetary compensation was provided for participation. However, to support the safe completion of the mountaineering activity, essential mountaineering apparel (including windproof jackets, windproof trousers, and down jackets) was provided as in-kind support. This support was uniformly provided to all participants and was not contingent on study completion or performance. Prior to completing the questionnaires, participants were provided with study information, which was considered informed consent, and they were free to withdraw from the study at any time without any consequences. The study protocol was approved by the Ethics Committee of China University of Geosciences, and all participants provided written informed consent prior to participation. The study was conducted in strict accordance with ethical guidelines.

**Table 2:** Participant characteristics.

Total (*n*)	Age (years)	Male	Female	Height (cm)	Weight (kg)	Experience above 5,000 m	Medications	Smoking history	High-altitude exposure in the past 6 months
15	28 ± 8	8	7	176 ± 11	71.3 ± 11.5	7	0	0	0

### 2.3 Acute mountain sickness scoring

The Lake Louise Score (LLS) is a self-report questionnaire used to assess symptoms such as headache, nausea or vomiting, fatigue and dizziness, with each item rated from 0 (absent) to 3 (severe), yielding a total score ranging from 0 to 12 (Roach et al., 2018). According to the diagnostic criteria, at altitudes above 2,500 m, the presence of headache plus at least one additional symptom and a total LLS score ≥3 is considered indicative of AMS.

### 2.4 Rating of perceived exertion

The Borg RPE scale was used to record perceived exertion three times per day after each trekking segment (Borg, 1990). This is a 15-point scale ranging from 6 to 20, where 6 represents “very, very light” exertion and 20 represents maximal exertion. Compared with simplified 0–10 versions, the 6–20 scale provides finer gradations, making it easier for participants to report their exertion during prolonged, long-distance mountaineering. Previous studies have shown that higher Borg RPE scores on trekking days are associated with increased AMS incidence and higher LLS scores on the following day (Mellor et al., 2014; Hazlerigg et al., 2016). Before the ascent, participants were familiarised with the 6–20 arbitrary units and their meaning.

### 2.5 Anxiety assessment

The State–Trait Anxiety Inventory (STAI) developed by (Spielberger, 1983) is a widely used self-report measure of anxiety (Karinen and Tuomisto, 2017). The inventory consists of 40 items, including 20 items assessing state anxiety (STAI-Y1) and 20 items assessing trait anxiety (STAI-Y2). Responses are given on a 4-point Likert scale indicating the intensity of feelings regarding statements such as “I feel calm” or “I feel tense,” ranging from “not at all” to “very much so.” Subscale scores range from 20 to 80, with higher scores indicating higher levels of anxiety. STAI-Y1 reflects transient tension and worry in the current situation, whereas STAI-Y2 reflects a relatively stable tendency toward anxiety. In the present study, STAI-Y1 was administered once before the climb and once after completion of the high-altitude ascent to compare changes in state anxiety.

### 2.6 Statistical analysis

All data were analysed using SPSS version 27.0. Because the sample size was less than 50, the Shapiro–Wilk test was used to assess normality before applying parametric procedures (Razali and Wah, 2011). The results of the Shapiro–Wilk tests (Table 3) were used to guide the selection of parametric or nonparametric statistical analyses for each outcome variable. For within-subject pre–post comparisons, paired-sample t tests were applied to variables that met the assumption of normality, whereas the Wilcoxon signed-rank test was used for variables that did not satisfy the normality assumption (Haslinger et al., 2018). For between-group comparisons based on AMS status, summit attainment, and sex, independent-sample t tests were employed for normally distributed variables, while the Mann–Whitney *U* test was used for variables with non-normal distributions (Habelt et al., 2023; Li et al., 2025a, 2025b). To explore factors associated with post-climb state anxiety, multiple linear regression analyses were conducted with post-climb STAI-Y1 scores as the dependent variable and pre-climb STAI-Y1 scores and changes in SpO₂ and RPE as independent variables. A two-sided *p* < 0.05 was considered statistically significant (Castanier et al., 2011).

**Table 3:** Normality tests for LLS, SpO₂, RPE, and STAI before and after the climb.

Category	Variable	Mean ± SD	Skewness	Kurtosis	Shapiro–Wilk test	
					W statistic	*p*
Pre	LLS	1.022 ± 1.581	1.565	1.582	0.717	0.000
	SpO₂	93.067 ± 2.281	−1.760	3.488	0.807	0.005
	RPE	6.667 ± 0.976	0.788	−1.615	0.603	0.000
	STAI	46.533 ± 12.933	0.000	−0.492	0.989	0.999
Post	LLS	5.822 ± 2.635	0.621	0.675	0.967	0.809
	SpO₂	87.177 ± 4.774	−1.371	2.663	0.893	0.075
	RPE	18.600 ± 0.828	−0.801	0.337	0.805	0.004
	STAI	43.933 ± 14.144	−0.177	−1.062	0.952	0.558

*p < 0.05, **p < 0.01, ***p < 0.001.

## 3 Results

All climbers arrived by vehicle at Haba Village (2,726 m) at 15:00 on the first day, where they purchased supplies, coordinated logistical support and completed the necessary formalities. On the second day, they departed from Haba Village and hiked with light packs to base camp. On the third day, they set out from base camp toward the summit of Mount Haba. To ensure safety and efficiency, camp construction, cooking and post-meal cleaning were handled by dedicated staff, while other tasks were carried out collaboratively by the research team and the club members.

**Figure 1:**
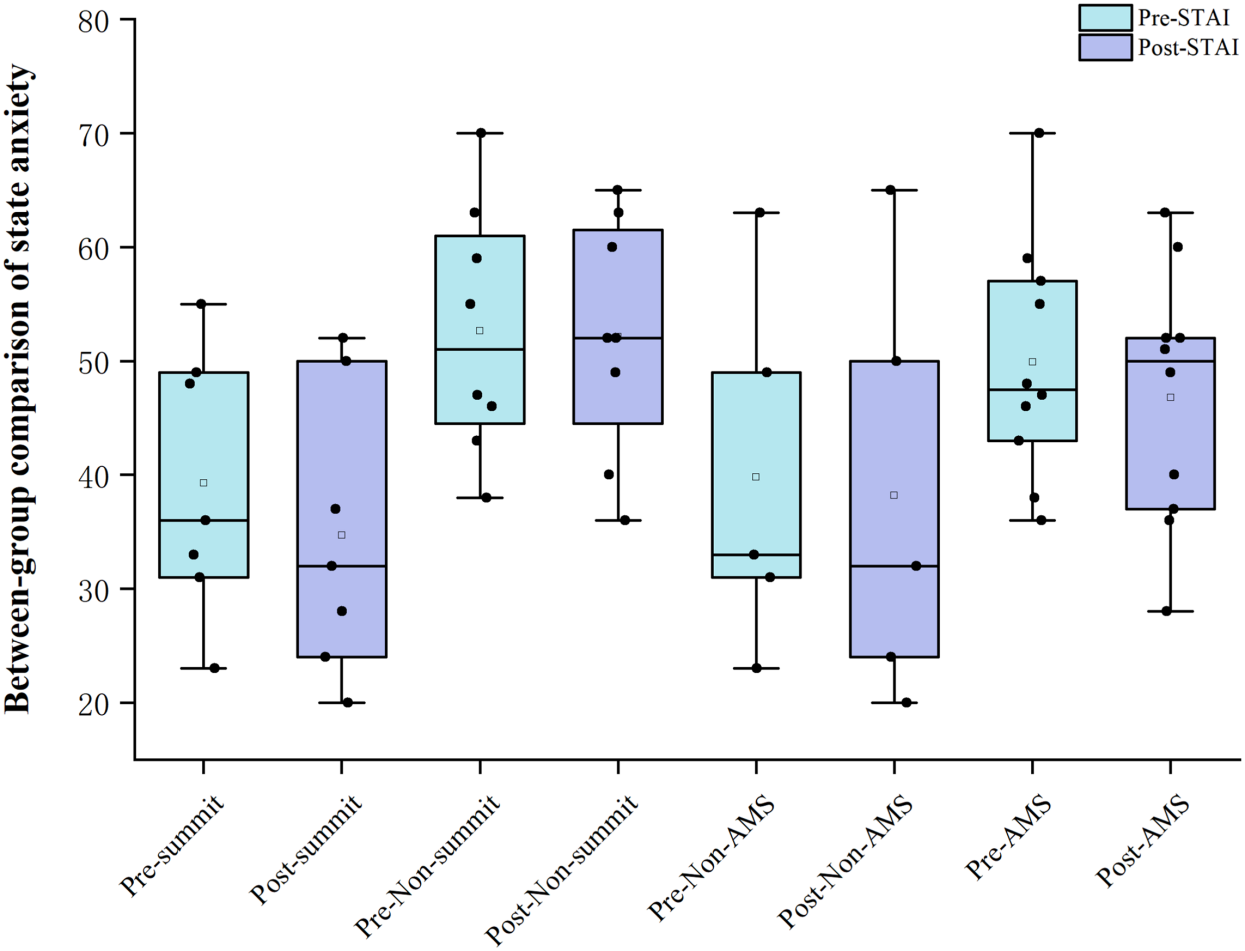
Between-group comparison of pre- and post-climb state anxiety.

During the three-day high-altitude ascent, continuous monitoring of the Lake Louise Score and oxygen saturation showed that four climbers developed acute mountain sickness on the first day, with one participant reaching an LLS score of 9. Over the course of the expedition, a total of 10 climbers experienced varying degrees of AMS, and the highest LLS score observed was 12; symptoms gradually diminished or resolved after descent. Notably, five climbers did not develop AMS at any time during the ascent. Figure 1 illustrates the pre- to post-climb changes in state anxiety between the two groups. With respect to summit success, eight climbers (53%) did not reach the summit, primarily due to insufficient physical strength (two turned back at 5,000 m, two at 4,700 m, one at 4,500 m and one at 4,400 m) or severe AMS (one turned back at 4,500 m and one at 4,250 m), and these withdrawals were made for safety reasons. It is noteworthy that among the seven climbers who reached the summit, five did not experience AMS at any point during the entire climb.

### 3.1 Changes before and after the climb

**Figure 2:**
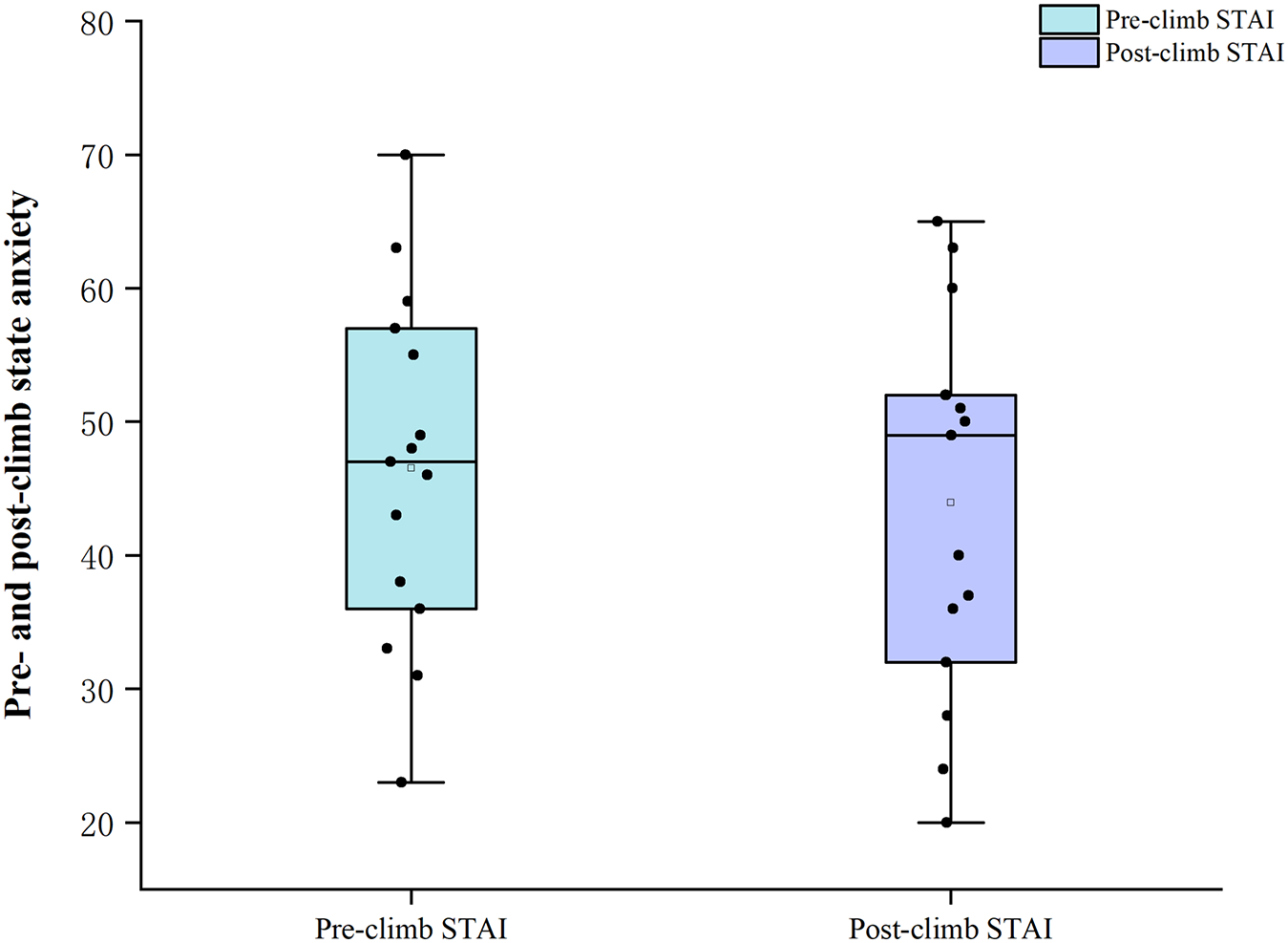
Pre- and post-climb state anxiety.

Baseline trait anxiety was 40.7 ± 6.2, and no significant sex differences were observed in demographic variables or baseline psychological characteristics. Compared with pre-climb values, the acute mountain sickness symptom score (LLS) increased from a median of 0.0 (0.0, 2.0) to 5.67 (4.30, 6.70) after the climb, and this change was statistically significant (*p* < 0.001). Arterial oxygen saturation (SpO₂) decreased significantly from 94.00 (91.70, 94.70)% before the climb to 87.67 (84.30, 91.00)% after the climb (*p* < 0.001). Similarly, the median rating of perceived exertion (RPE) increased from 6.00 (6.00, 8.00) before the climb to 19.00 (18.00, 19.00) after the climb (*p* < 0.001; Razali and Wah, 2011). By contrast, state anxiety scores (STAI-Y1) showed a slight decrease after the climb (from 46.5 ± 12.9 before the climb to 43.9 ± 14.1 afterwards; Figure 2), but this change was not statistically significant (*p* = 0.603; Tables 3, 4).

**Table 4:** Tests of differences in LLS, SpO₂, RPE, and STAI before and after the climb.

Test variable	Pre- vs. post-climb		*z*/*t* value	*p*
	Before the climb (*n* = 15)	After the climb (*n* = 15)		
LLS	0.000 (0.0,2.0)	5.670 (4.3,6.7)	−4.254	0.000***
SpO₂	94.000 (91.7,94.7)	87.670 (84.3,91.0)	−3.864	0.000***
RPE	6.000 (6.0,8.0)	19.000 (18.0,19.0)	−4.836	0.000***
STAI	46.53 ± 12.93	43.93 ± 14.14	0.525	0.603

*p < 0.05, **p < 0.01, ***p < 0.001.

### 3.2 Between-group comparisons

#### 3.2.1 Participants with and without AMS

Compared with participants who did not develop AMS (Table 5), those in the AMS group had significantly higher LLS scores both before and after the climb (pre-climb median 0.8 vs. 0.0; post-climb 6.90 ± 2.37 vs. 3.67 ± 1.72; both *p* < 0.05). There were no significant between-group differences in SpO₂ or RPE at either time point, nor in their change scores (all *p* > 0.05). For STAI-Y1, baseline scores were significantly higher in the AMS group (49.9 ± 10.4 vs. 39.8 ± 16.0; *p* < 0.05). For STAI-Y1, baseline scores in the AMS group (49.9 ± 10.4) tended to be higher than those in the non-AMS group (39.8 ± 16.0), although this difference did not reach statistical significance (*p* = 0.161).

**Table 5:** Distribution of LLS, SpO₂, RPE, and STAI before and after the climb by AMS status.

Test variable	Time point	AMS		*z*/*t* value	*p*
		AMS− (*n* = 5)	AMS+ (*n* = 10)		
LLS	Pre	0.000 (0.0,0.0)	0.835 (0.0,3.1)	−2.325	0.020*
	Post	3.67 ± 1.72	6.90 ± 2.37	−2.696	0.018*
SpO₂	Pre	93.330 (92.5,94.7)	94.000 (90.9,94.8)	−0.123	0.902
	Post	89.40 ± 3.66	86.06 ± 5.04	1.307	0.214
RPE	Pre	6.000 (6.0,6.0)	7.000 (6.0,8.0)	−1.871	0.061
	Post	18.000 (17.5,19.0)	19.000 (18.8,19.0)	−1.459	0.145
STAI	Pre	39.80 ± 16.04	49.90 ± 10.40	−1.486	0.161
	Post	38.20 ± 18.90	46.80 ± 11.18	−1.12	0.283

#### 3.2.2 Successful versus unsuccessful summiters

With respect to summit success, non-summiters showed significantly higher state anxiety than summiters, both before the climb (52.9 vs. 39.3, *p* < 0.05) and after the climb (52.0 vs. 34.7, *p* < 0.05), with STAI-Y1 scores consistently higher in the non-summit group. No significant between-group differences were observed in LLS, SpO₂ or RPE (Table 6).

**Table 6:** Distribution of LLS, SpO₂, RPE, and STAI before and after the climb by summit status.

Test variable	Time point	Summit status		*z*/*t* value	*p*
		Non-summiters (*n* = 8)	Summiters (*n* = 7)		
LLS	Pre	0.835 (0.1,2.8)	0.000 (0.0,0.0)	−1.883	0.06
	Post	6.92 ± 2.88	4.57 ± 1.76	1.868	0.084
SpO₂	Pre	94.165 (91.8,94.9)	93.330 (91.7,94.7)	−0.757	0.449
	Post	86.16 ± 6.03	88.33 ± 2.81	−0.871	0.4
RPE	Pre	6.000 (6.0,8.0)	6.000 (6.0,8.0)	−0.354	0.724
	Post	18.88 ± 0.64	18.29 ± 0.95	1.425	0.178
STAI	Pre	52.88 ± 11.03	39.29 ± 11.56	2.329	0.037*
	Post	52.00 ± 10.45	34.71 ± 12.39	2.933	0.012*

#### 3.2.3 Sex differences

**Figure 3:**
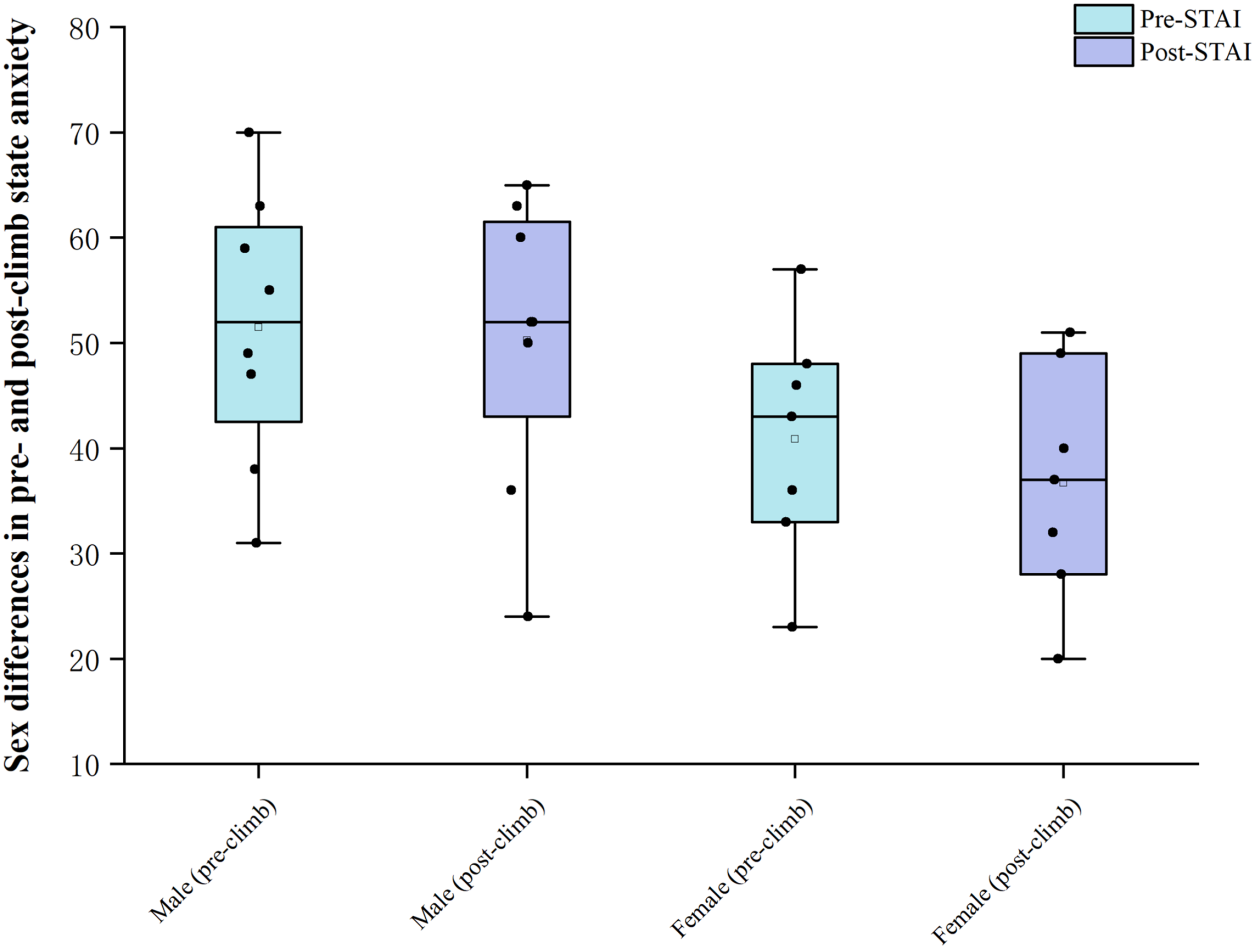
Sex differences in pre- and post-climb state anxiety.

With respect to sex, Figure 3 shows that male and female participants had similar distributions of STAI-Y1 scores before and after the climb, with a slight decrease after the climb in both groups and only small absolute changes. At all time points, there were no significant sex differences in LLS, SpO₂, RPE or STAI-Y1 (Table 7).

**Table 7:** Distribution of LLS, SpO₂, RPE, and STAI before and after the climb by sex.

Test variable	Time point	Sex		*z*/*t* value	*p*
		Male (*n* = 8)	Female (*n* = 7)		
LLS	Pre	1.000 (0.0,3.2)	0.000 (0.0,0.7)	−1.004	0.315
	Post	6.58 ± 3.38	4.95 ± 1.11	1.217	0.245
SpO₂	Pre	92.46 ± 2.85	93.76 ± 1.27	−1.114	0.285
	Post	90.835 (86.5,91.2)	86.000 (83.7,88.3)	−1.737	0.082
RPE	Pre	6.000 (6.0,7.5)	6.000 (6.0,8.0)	−0.707	0.48
	Post	19.000 (18.0,19.0)	19.000 (17.0,19.0)	−0.394	0.694
STAI	Pre	51.50 ± 12.94	40.86 ± 11.16	1.693	0.114
	Post	50.25 ± 14.01	36.71 ± 11.13	2.05	0.061

### 3.3 Analysis of factors associated with state anxiety

Multiple regression analysis (Table 8) indicated that the model linking pre- and post-climb state anxiety was significant, with a coefficient of determination *R*^2^ = 0.920, suggesting that the independent variables together accounted for approximately 92.0% of the variance in post-climb state anxiety (*F* = 42.115, *p* < 0.001). Pre-climb state anxiety (STAI_Pre) was strongly and positively associated with post-climb state anxiety, with an unstandardised coefficient B = 1.002 (standardised *β* = 0.916, *p* < 0.001), indicating that each 1-point increase in baseline state anxiety was accompanied by an approximately 1.002-point increase in post-climb state anxiety. The change in perceived exertion during the ascent (ΔRPE) was also significantly and positively associated with post-climb state anxiety (B = 2.798, *p* < 0.05), indicating that participants with larger increases in RPE tended to report higher post-climb state anxiety. By contrast, the regression coefficient for change in oxygen saturation (ΔSpO₂) was negative (B = −0.380) but not statistically significant (*p* > 0.05), suggesting that the degree of SpO₂ decline did not exert a significant independent influence on post-climb state anxiety.

**Table 8:** Linear regression analysis of factors associated with state anxiety in graduate student climbers (*n* = 15).

Independent variables	Unstandardised coefficients		Standardised coefficients	*t*-value	*p*	Collinearity diagnostics	
	B	Standard error	Beta			VIF	Tolerance
Constant	−38.315	15.29	-	−2.506	0.029*	-	-
State anxiety STAI_Pre	1.002	0.098	0.916	10.222	0.000***	1.103	0.907
△SpO₂	−0.38	0.355	−0.104	−1.07	0.307	1.306	0.765
△RPE	2.798	1.165	0.23	2.402	0.035*	1.26	0.794
*R* ^2^	0.92						
Adjusted *R*^2^	0.898						
*F*	*F* = 42.115, *p* = 0.000						
D–W value	2.407						

Dependent variable, post-climb state anxiety (STAI_Post). *p < 0.05, **p < 0.01, ***p < 0.001.

## 4 Discussion

In this real-world high-altitude mountaineering setting, we found that overall state anxiety (STAI-Y1) showed a slight decrease after the climb, with a more pronounced reduction among participants who successfully reached the summit. Participants who developed AMS had higher pre-climb state anxiety than those without AMS; although their anxiety levels decreased after the climb, they remained relatively elevated and the between-group difference was not statistically significant. These findings suggest that pre-ascent anxiety is related to subsequent high-altitude responses and psychological burden. Previous work has shown that higher pre-ascent anxiety predicts both the occurrence and severity of later AMS (Boos et al., 2018), which is consistent with the pattern observed in the present study at the level of state anxiety.

We consider the main contribution of this study to lie in the following aspects. First, in a relatively uncommon sample of graduate student mountaineers, we used a pre–post design and integrated arterial oxygen saturation, perceived exertion, state anxiety and LLS into a single analytical framework to characterise the overall impact of high-altitude mountaineering on both physiological and psychological indices. Second, through multiple regression modelling, we demonstrated independent contributions of baseline state anxiety and the change in perceived exertion (ΔRPE) to post-climb state anxiety, whereas the influence of changes in oxygen saturation (ΔSpO₂) appeared comparatively limited.

### 4.1 Overall pattern of physiological load and state anxiety

Although high-altitude hypoxia affects climbers’ subjective somatic state (Zhou et al., 2025), this factor alone does not constitute a sufficient condition for producing transient mental-state disturbances (Ruffini and Cera, 2020), a finding that is consistent with our initial hypothesis. Our results showed that, despite a marked decrease in SpO₂ and a pronounced increase in RPE, state anxiety (STAI-Y1) actually declined after the climb. This pattern suggests that, while confronting substantial physiological stress, climbers may at the same time obtain a psychologically beneficial regulatory effect through increased feelings of achievement or self-efficacy. Some studies have reported that rising altitude tends to exert negative effects on mood (Shukitt-Hale et al., 1990; Kious et al., 2018; Wang et al., 2022; Özen and Vatansever, 2016; Kürkcü Akgönül et al., 2021). In contrast, our comparison of pre- and post-climb data indicated a downward trend in state anxiety, which may be related to the sense of accomplishment and psychological satisfaction associated with completing the ascent. Previous research has shown that, although individuals engaged in high-risk sports are prone to emotion-regulation difficulties in everyday life, activities such as mountaineering can strengthen self-regulatory capacities and reduce anxiety risk (Habelt et al., 2023). This is noteworthy given that mountaineering entails the possibility of severe injury or death (Gasser, 2022; Krieger et al., 2022; Muthomi et al., 2023) and often involves sustained effort over many hours, days or even weeks (Li et al., 2025a). However, in the present study, the observed reduction in state anxiety should be interpreted strictly as an empirical pattern rather than as evidence of a specific psychological mechanism, as this process was not directly assessed.

### 4.2 Individual differences in state anxiety across participant subgroups

Between-group comparisons further highlighted how state anxiety differed across individual characteristics. Pre-ascent state anxiety levels were higher in the AMS group than in the non-AMS group; however, this difference was not statistically significant (*p* = 0.161). State anxiety decreased after the ascent in both groups, with no significant between-group differences observed. These findings suggest that baseline state anxiety exhibited a non-significant tendency toward higher values among participants who developed AMS, rather than a meaningful group difference. Similar non-significant trends have been reported in previous high-altitude studies (Bärtsch and Swenson, 2013; Boos et al., 2018), but the underlying psychological mechanisms require further investigation using larger samples and direct measurements. In contrast, participants who reached the summit had significantly lower state anxiety than non-summiters both before and after the climb, implying that task completion and enhanced self-efficacy may buffer anxiety levels. This pattern aligns with previous models in which task completion is associated with increased self-efficacy and, in turn, lower anxiety (Brymer and Schweitzer, 2013; Lawrence et al., 2014), and suggests that goal-directed engagement during mountaineering may foster positive cognitive reappraisal and adaptive emotion regulation. Although no significant sex differences were observed in the present study, prior meta-analytic evidence indicates that women have a higher susceptibility to AMS than men (RR = 1.24, 95% CI 1.09–1.41; Hou et al., 2019). The discrepancy between our findings and earlier work may be related to sample characteristics and the relatively short duration of exposure in this study, which may have attenuated potential sex-related physiological differences.

### 4.3 Key factors associated with state anxiety

The multiple regression analysis further clarified the main factors associated with post-climb state anxiety. Pre-climb state anxiety emerged as the strongest correlate of post-climb state anxiety (*β* = 0.916, *p* < 0.001), indicating that baseline state anxiety exerts a sustained influence on subsequent psychological responses. The change in perceived exertion (ΔRPE) was also a significant positive factor (*p* < 0.05), whereas the change in oxygen saturation (ΔSpO₂) showed a negative but non-significant association, suggesting that hypoxia-related physiological indices make only a limited independent contribution to changes in state anxiety.

Existing empirical work supports this pattern. In a study of women undergoing an acute exercise bout, pre-exercise state anxiety accounted for approximately 27% of the variance in pre–post anxiety change (*p* < 0.001; Guszkowska, 2009), underscoring the strong and enduring influence of baseline emotional state on subsequent psychological responses. This relationship is particularly evident during high-intensity physical activity, where initial anxiety levels shape participants’ cognitive appraisal of challenging situations and their emotion-regulation processes (Castanier et al., 2011; Sołtysik et al., 2019). Moreover, perceived exertion (RPE) not only reflects physiological load but is also closely linked to psychological state. Studies have demonstrated significant associations between RPE and physiological markers such as electromyographic activity and heart rate variability, and increases in RPE during exercise are often accompanied by heightened feelings of anxiety (Hollander et al., 2003). In high-intensity or prolonged exercise settings, rising RPE tends to coincide with elevated body temperature and increasing metabolic stress, which may further amplify anxiety (Tucker, 2009).

Taken together, although physical load increased markedly during the ascent, overall state anxiety among the graduate students did not rise and instead showed a slight decrease after the climb, in contrast to findings from many experimental studies of hypoxia. Participants who successfully reached the summit tended to exhibit more stable mood and greater attentional focus. In real high-altitude environments, task completion and the accompanying sense of achievement among graduate students may help buffer state anxiety responses.

## 5 Conclusion

As a form of high-load exercise performed in an extreme environment, short-term high-altitude mountaineering appeared to exert a beneficial effect on state anxiety regulation among participants; however, changes in state anxiety scores were not statistically significant. However, when designing questionnaires for mountaineers, additional contextual dimensions such as grit, sleep, and perceived environmental stress could be incorporated to more comprehensively characterize psychological responses in high-altitude environments.

## 6 Limitations

The mountaineering period in the present study was relatively short, the sample size was small (*N* = 15), and several subgroups in the sex- and AMS-stratified analyses were limited in size; these factors may have introduced bias and reduced statistical power. In addition, state anxiety was assessed only before and after the climb, which may have resulted in potential measurement timing bias. Future research should consider longer expeditions and more diverse samples, combined with dynamic monitoring via wearable devices, to more accurately characterise the temporal dynamics of emotional changes during mountaineering.

## Data Availability

The original contributions presented in the study are included in the article/supplementary material, further inquiries can be directed to the corresponding author.
